# METRIC-GUIDED IMAGE RECONSTRUCTION BOUNDS VIA CONFORMAL PREDICTION

**Published:** 2024-07-02

**Authors:** Matt Y. Cheung, Tucker J. Netherton, Laurence E. Court, Ashok Veeraraghavan, Guha Balakrishnan

**Affiliations:** Department of Electrical & Computer Engineering, Rice University, Houston TX; Department of Radiation Physics, The University of Texas M.D. Anderson Cancer Center, Houston TX; Department of Radiation Physics, The University of Texas M.D. Anderson Cancer Center, Houston TX; Department of Electrical & Computer Engineering, Rice University, Houston TX; Department of Electrical & Computer Engineering, Rice University, Houston TX

**Keywords:** Uncertainty, Calibration, Inverse Problems

## Abstract

Recent advancements in machine learning have led to the development of novel medical imaging systems and algorithms that address ill-posed problems. Assessing their trustworthiness and understanding how to deploy them safely at test time remains an important and open problem. In this work, we propose using conformal prediction to compute valid and distribution-free bounds on downstream metrics given reconstructions generated by one algorithm, and retrieve upper/lower bounds and inlier/outlier reconstructions according to the adjusted bounds. Our work offers 1) test time image reconstruction evaluation without ground truth, 2) downstream performance guarantees, 3) meaningful upper/lower bound reconstructions, and 4) meaningful statistical inliers/outlier reconstructions. We demonstrate our method on post-mastectomy radiotherapy planning using 3D breast CT reconstructions, and show 1) that metric-guided bounds have valid coverage for downstream metrics while conventional pixel-wise bounds do not and 2) anatomical differences of upper/lower bounds between metric-guided and pixel-wise methods. Our work paves way for more meaningful and trustworthy test-time evaluation of medical image reconstructions. Code available at https://github.com/matthewyccheung/conformal-metric.

## 1 Introduction

Image reconstruction is a crucial component of many clinical workflows. From computed tomography (CT) to magnetic resonance imaging (MRI), many medical imaging modalities are constructed by combining observed signals of patient anatomy into one “complete” image. For example, traditional CT reconstruction algorithms take thousands of 2D x-ray observations around a patient’s anatomy as input, and “backproject” these signals to produce 3D CT volumes [1]. These CT scans may in turn be used by radiotherapy treatment (RT) software to produce dosage plans for cancer treatment [2]. Image reconstruction accuracy and reliability can therefore have direct impacts on patient standard-of-care.

Often times, particularly in scenarios with limited imaging capabilities such as low-resource clinics [3, 4, 5], these reconstruction algorithms work with “sparse” observations, i.e., those that do not contain all information of the anatomy of interest. For example, sparse CT algorithms use limited (< 100 instead of the standard 100s) 2D x-ray observations to generate 3D CT scans [2, 6, 7]. In sparse image reconstruction, the observed information is not sufficient to recover the true image with complete certainty. Uncertainty originates from multiple sources in the reconstruction pipeline, including variations in image acquisition settings to quantum noise. To tackle uncertainty, classical reconstruction algorithms use human-defined priors, such as sparsity constraints [8], to choose one likely solution from the potentially infinite reconstructions consistent with the observations. While long successful, this approach does not describe the space of plausible reconstructions, and therefore, offers no insight into the resulting uncertainty in downstream clinical metrics which rely on those reconstructions, e.g., predicted radiation dose to organs in RT and organ volume.

Modern deep neural network (DNN) reconstruction algorithms, which learn complex priors from large datasets, offer the exciting alternative of returning multiple plausible reconstruction samples per input. Popular approaches to do so include deep generative modeling [9, 10], and randomized parameter initialization/dropout [11, 12]. A simple yet powerful way to use such samples is to compute *confidence bounds* on downstream clinical metrics. For example, in RT planning, confidence bounds can specify the range of doses to an organ, giving radiation physicists a better sense of the risks of the procedure.

While promising, there is a critical issue with using such bounds naively: if there are inherent biases of the reconstruction DNN, the resulting bounds will be misspecified. Indeed, DNNs are well-known to suffer from confidently incorrect predictions [13, 14], and in the context of generative tasks these are often termed “hallucinations” [15]. Examples of reconstruction hallucinations include systematically higher intensities for certain regions and certain organ geometry. These biases can degrade, and in the worst case, render confidence bounds on downstream metrics completely useless. Hence, there is a pressing need for methods to produce *calibrated prediction bounds* from DNN generative models, i.e., bounds that contain the ground truth metrics with certainty for new observations.

Recently, conformal prediction (CP) has gained acceptance as a powerful technique to calibrate uncertainty bounds of values (either categorical or continuous) predicted by a machine learning (ML) algorithm [16, 17, 18, 19, 20]. One approach is split conformalized quantile regression [21]. The main idea is to use a “calibration set” of data samples with ground truth target values to learn a compensation factor with which we may adjust bounds returned by the ML algorithm. Under the assumption of exchangeability, the resulting bounds will provably, on average, contain the ground truth target values (1 − *α*)% of the time for some set miscoverage rate *α* (e.g., often set to 0.05 or 0.10). However, while successful on a variety of traditional ML tasks such as image classification, the extension to image reconstruction problems is unnatural due the high-dimensional nature of the outputs.

In this work, we propose using CP to compute valid and distribution-free bounds on downstream metrics given images generated by an image reconstruction algorithm. Assuming a calibration set of reconstructions with true metric annotations (e.g., RT dose values to specific organs) as input, we use CP to adjust estimated quantiles of downstream metrics and return meaningful 1) upper/lower bound reconstructions and 2) statistical inliers/outliers.

We focus our empirical evaluations on post-mastectomy RT planning using 3D breast CT reconstructions, and show that 1) that metric-guided bounds have valid coverage for downstream metrics while conventional pixel-wise bounds do not and 2) anatomical differences of upper/lower bounds between metric-guided and pixel-wise methods.

Our work paves way for more meaningful and trustworthy evaluation of medical image reconstructions at test time.

### 1.1 Related Work

#### 1.1.1 Conformal Prediction

Conformal prediction (CP) is a model-agnostic and distribution-free approach to attain valid prediction sets under the assumption of exchangeability [17, 18, 19, 20]. A common CP approach is split CP [16]. Based on a calibration dataset $\left\{\left({\overset{ˆ}{Y}}^{1},Y^{1}),\ldots ,({\overset{ˆ}{Y}}^{n},Y^{n}\right)\right\}$, we can construct a prediction set $C(\overset{ˆ}{Y})$ for a fresh test point $Y^{n+1}$, such that:

(1) $$P[Y^{n+1}\in C({\overset{ˆ}{Y}}^{n+1})]\geq 1-\alpha$$

where *α* is a user-specified miscoverage rate. This setup provides marginal coverage - on average, the prediction sets contain ground truth (1−α)% of the time. For regression tasks, the prediction set is an interval [22]. Longer lengths indicate higher uncertainty. To derive the prediction intervals, the key idea is to use residuals from a calibration set as a conformity scores, s, to compute upper and lower bounds [17, 18, 19, 20]. For symmetric adjustments, the (1−α)th quantile of the conformity scores is used to compute the prediction interval. For asymmetric adjustments, the conformity scores are computed separately for upper and lower adjustments. Many conformity scores exist, including absolute residual scores [22] and quantile-based scores [21]. Our work uses quantile-based conformity scores [21] to adjust estimated quantiles of downstream metrics from reconstruction samples to achieve valid prediction intervals.

#### 1.1.2 Conformal Prediction for Image-to-Image Regression Problems

Finding quantiles of high-dimensional data like images is a difficult problem because there are an infinite number of (1−α)-quantiles and are only unique when a direction is specified [23, 24]. How do we pick such a direction? The conventional (pixel-wise) method is to pick the direction where all pixels are independent [25, 26, 27]. Recent work in CP provide upper and lower bounds based on pixel intensity [28, 29, 30] and principal components [31]. While pixel-wise prediction intervals are easy to interpret, they do not consider spatial correlations. While using principal components considers spatial correlations, it does not capture meaningful and practical uncertainty for downstream processes and is prohibitively costly to compute for large images. Furthermore, both methods provide upper and lower bounds not sampled from the learned manifold, yielding implausible images. An interesting approach is to calibrate the upper and lower bounds in the direction of semantic features [32]. However, this method requires training a generative model with disentangled latent spaces. Our work uses downstream metrics for calibration and retrieves image reconstruction bounds and prediction sets directly from the set of sampled reconstructions.

## 2 Method

**Figure 1:**
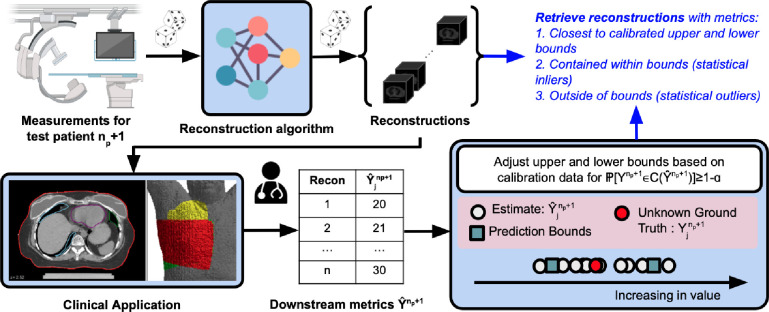
Overview of our approach. Assume probabilistic measurement and reconstruction processes, $n_{p}$ patients for calibration, and 1 patient for testing. For test patient “$n_{p}+1$” with unknown ground truth reconstruction and metric, 1) acquire measurements, 2) attain a set of reconstructions, 3) extract downstream metrics, 4) adjust upper and lower bounds of metric based on a calibration procedure, and 5) retrieve reconstructions with metrics closest to the calibrated upper and lower bounds, contained within bounds (statistical inliers), and outside of bounds (statistical outliers).

We consider a 3-D reconstruction setting for a downstream application with a chosen downstream metric. The measurement and reconstruction algorithms are assumed to be probabilistic. We follow the split conformal prediction procedure [16] by using $n_{p}$ patients for the calibration dataset and 1 test patient $n_{p}+1$ with unknown ground truth volume and metric as the test dataset. For each patient i in the calibration dataset, we reconstruct a set of volumes ${\overset{ˆ}{V}}^{i}={\left\{{\overset{ˆ}{V}}_{j}^{i}\right\}}_{j=1}^{n_{r}}$ of size $n_{r}$. Each patient’s reconstructed volumes are used to attain a set of estimated metrics ${\overset{ˆ}{Y}}^{i}={\left\{{\overset{ˆ}{Y}}_{j}^{i}\right\}}_{j=1}^{n_{r}}$. Each patient’s ground truth volume is used to attain a ground truth metric $Y^{i}$. For the test patient, we reconstruct a set of volumes ${\overset{ˆ}{V}}^{n_{p}+1}={\left\{{\overset{ˆ}{V}}_{j}^{n_{p}+1}\right\}}_{j=1}^{n_{r}}$ and estimate metrics ${\overset{ˆ}{Y}}^{n_{p}+1}={\left\{{\overset{ˆ}{Y}}_{j}^{n_{p}+1}\right\}}_{j=1}^{n_{r}}$. Assuming $\left({\overset{ˆ}{Y}}^{i},Y^{i}\right)$ for $i=1,\ldots ,n_{p}+1$ are exchangeable, we find the prediction interval satisfying the marginal coverage guarantee in Eq. 1 using a quantile-based conformity score $s^{i}=max[Q_{\alpha /2}({\overset{ˆ}{Y}}^{i})-Y^{i},Y^{i}-Q_{1-\alpha /2}({\overset{ˆ}{Y}}^{i})]$ [21]. The prediction interval is given as $C({\overset{ˆ}{Y}}^{n_{p}+1})=[Q_{\alpha /2}({\overset{ˆ}{Y}}^{n_{p}+1})-q,Q_{1-\alpha /2}({\overset{ˆ}{Y}}^{n_{p}+1})+q]$ where q is the (1−α)th quantile of the conformity scores with a finite sample correction. Asymmetric bounds can be used as well, where the upper and lower bounds are adjusted separately. We retrieve the volumes 1) closest to the upper and lower bounds of the prediction intervals $\left[{\overset{ˆ}{V}}_{LB}^{n_{p}+1},{\overset{ˆ}{V}}_{UB}^{n_{p}+1}\right]$ based on the $L_{1}$ norm, 2) contained within the prediction intervals (inliers), and 3) outside the prediction intervals (outliers). We provide an overview in Fig. 1 and pseudo-code for symmetric bounds in Algorithm 1.

**Algorithm 1:** Metric-guided Retrieval (Symmetric Bounds)

▷ Perform calibration to get upper and lower bound adjustment
**for** $i=1:n_{p}$ **do**
$s_{i}=max[Q_{\alpha /2}({\overset{ˆ}{Y}}^{i})-Y^{i},Y^{i}-Q_{1-\alpha /2}({\overset{ˆ}{Y}}^{i})]$
**end for**
$q=Q_{\frac{\left[\left(n_{p}+1)(1-\alpha \right)\right]}{n_{p}}}(s)$
▷ Compute prediction interval for patient in test dataset
$C({\overset{ˆ}{Y}}^{n_{p}+1})=[LB({\overset{ˆ}{Y}}^{n_{p}+1}),UB({\overset{ˆ}{Y}}^{n_{p}+1})]=[Q_{\alpha /2}({\overset{ˆ}{Y}}^{n_{p}+1})-q,Q_{1-\alpha /2}({\overset{ˆ}{Y}}^{n_{p}+1})+q]$
▷ Retrieve upper and lower bound reconstructions
${\overset{ˆ}{V}}_{LB}^{n_{p}+1}={\arg min\;}_{{\overset{ˆ}{V}}_{j}^{n_{p}+1}}\;|{\overset{ˆ}{Y}}_{j}^{n_{p}+1}-LB({\overset{ˆ}{Y}}^{n_{p}+1})|$
${\overset{ˆ}{V}}_{UB}^{n_{p}+1}={\arg min\;}_{{\overset{ˆ}{V}}_{j}^{n_{p}+1}}\;|{\overset{ˆ}{Y}}_{j}^{n_{p}+1}-UB({\overset{ˆ}{Y}}^{n_{p}+1})|$
▷ Retrieve inliers and outlier reconstructions
$Inliers\;=\{{\overset{ˆ}{V}}_{j}^{n_{p}+1}\in {\overset{ˆ}{V}}^{n_{p}+1}|{\overset{ˆ}{Y}}_{j}^{n_{p}+1}\in [LB({\overset{ˆ}{Y}}^{n_{p}+1}),UB({\overset{ˆ}{Y}}^{n_{p}+1})]\}$
$Outliers\;=\{{\overset{ˆ}{V}}_{j}^{n_{p}+1}\in {\overset{ˆ}{V}}^{n_{p}+1}|{\overset{ˆ}{Y}}_{j}^{n_{p}+1}\notin [LB({\overset{ˆ}{Y}}^{n_{p}+1}),UB({\overset{ˆ}{Y}}^{n_{p}+1})]\}$

To verify the retrieved images are representative of the bounds at test time, we compute retrieval error defined as:

(2) $$\varepsilon_{B}=\frac{{\overset{ˆ}{Y}}_{B}^{n_{p}+1}\;-\;B^{n_{p}+1}}{UB^{n_{p}+1}\;-\;LB^{n_{p}+1}}\times 100\%$$

where B denotes the calibrated bound and can be upper bound UB or lower bound LB, and ${\overset{ˆ}{Y}}_{B}^{n_{p}+1}=arg\;{min}_{{\overset{ˆ}{Y}}_{j}^{n_{p}+1}}\;|{\overset{ˆ}{Y}}_{j}^{n_{p}+1}-B^{n_{p}+1}|$ are the estimated metrics closest to the calibrated bounds.

## 3 Experiments

Radiotherapy Planning: We use the Radiation Planning Assistant (RPA, FDA 510(k) cleared), a web-based tool for radiotherapy planning. [3, 4, 5]. RPA automates treatment planning on CT images and provides dose and plan reports for clinics in low-and-middle-income countries [3, 4, 5]. Dose statistics specify what percentage of organ volume receives a particular dose. Structural statistics are from organ segmentation and specify metrics such as organ volume and Hausdorff distance [33]. We use a dose prescription of 25 fractions in 50Gy (2.00Gy/fraction) for supraclavicular (SCV) and tangential field irradiation. The RPA automatically segments organs at risk and then applies a single-isocenter technique with matched tangential and SCV fields to treat the chest wall and SCV region.

### Dataset:

We use a de-identified CT dataset of 20 patients retrospectively treated with radiotherapy at The University of Texas MD Anderson Cancer Center. All CT images were of patients who had received surgical mastectomy to the right side of the body, and radiotherapy to the post-mastectomy chest wall and/or axillary lymph nodes. This research was conducted using an approved institutional review board protocol. Each ground truth CT is of size (512 × 512× Number of slices). For each patient, we generate 10 digitally reconstructed radiographs (DRR) from the ground truth CT scan using the TIGRE toolbox [34]. The DRRs simulate image acquisition from a cone-beam geometry. We simulate physical randomness (beam angle variability and sensor noise) by generating DRRs with 3% noise and 50 random projections between 0 and 360 degrees. While simulating beam angle variability and sensor noise does not encompass all sources of randomness in practice, our method remains theoretically robust without requiring modifications to address additional factors of noise and bias. The number of projections was increased from 2 to 50 until organ boundaries were perceptually discernible in the reconstruction by the RPA. Because this work aims to showcase the feasibility of CP for image reconstruction, we assume that such a low-cost sparse CT device will be created in future work that gives acceptable reconstruction image quality. We use a self-supervised model, Neural Attenuation Fields (NAF), for reconstruction [35]. Each reconstruction is uncropped and contains the full scan. We use the default parameter setting in NAF [35] and introduce computational randomness through random initializations of NAF [36, 11]. Ultimately, we construct a dataset of 20 patients with 10 reconstructions each. To construct the conventional pixel-wise upper and lower bounds, we take each individual pixel’s upper and lower quantile intensities.

#### Validation:

We validate our method by assessing coverage, interval length, and number of outliers for our metric-guided method with symmetric and asymmetric adjustments, and the conventional pixel-wise method. Coverage is defined as the fraction of patients with ground truth metrics within the bounds. For metric-guided bounds, we use leave-one-out cross-validation on 20 patients and report metrics: volume of ipsilateral lung that received 20Gy (Right Lung $V_{20}$), maxmimum dose to the heart (Heart $D_{0}$), and dose that 35% volume of the ipsilateral lung receives (Right Lung $D_{35}$). For conventional pixel-wise bounds, we compute the coverage over all patients and process the pixel-wise upper and lower bounds in RPA to attain dose and structural statistics. We use the finite sample correction $(1-\alpha {)}_{adj}=\frac{\left\lceil \left(n_{p}+1\right)(1-\alpha )\right]}{n_{p}}$ [21, 20] for target coverage of $[(1-\alpha {)}_{adj}]\%$. If the lower bound is greater than the upper bound, the prediction interval size is zero and does not contain the ground truth.

## 4 Results

Our results are presented in Table. 1. We observe that while the average interval length of pixel-wise methods is smaller, pixel-wise methods suffer from significant undercoverage. In some cases like example Right Lung Volume, the average interval length is zero. This is because pixel-wise upper bounds produce smaller volumes than lower bounds. On the other hand, metric-guided bounds produce valid coverage. Our metric-guided method with symmetric adjustments tend to perform better than asymmetric adjustment (except for Right Lung Volume). We also show the probability of a reconstruction classified as outliers based on the bounds. While symmetric and asymmetric bounds perform similarly for Heart $D_{0}$, Heart Volume and Right Lung $V_{20}$, asymmetric bounds are more effective at identifying outliers for Right Lung Volume. We suspect these observations where asymmetric bounds are more effective than symmetric bounds are due to model misspecification (i.e. high estimation bias) since the reconstruction algorithm does not directly optimize for downstream metrics.

### 4.1 Interpreting Pixel-wise and Metric-guided Bounds

**Figure 2:**
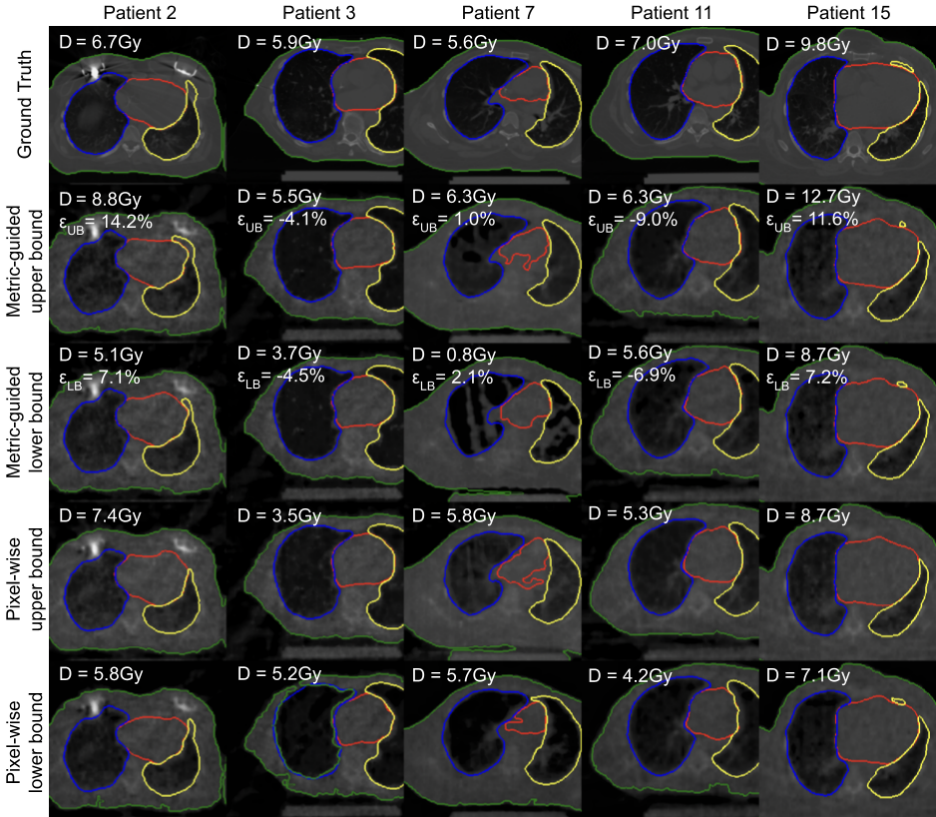
Metric-guided bounds account for spatial correlations that affect downstream metrics. For maximum dose to the heart (D) with target coverage of 90%, we show contours for heart (red), right lung (blue), left lung (yellow), and body (green) overlaid on CT slices. Pixel-wise upper and lower bounds differ in pixel-wise intensity, while metric-guided bounds differ in the spatial distribution of pixel intensities. Pixel-wise upper bounds have larger heart volumes than lower bounds, while metric-guided bounds have similar heart volumes. Retrieval error $\varepsilon_{B}$ is the difference between estimated and actual bound divided by the interval length (Eq. 2). We used symmetric adjustments for metric-guided bounds.

We show that the upper/lower bounds can be interpretated (Fig. 2). We find that pixel-wise upper and lower bounds are perceptually similar and only differ in their intensity, while metric-guided bounds differ in the spatial distribution of pixel intensities. This indicates that metric-guided bounds take spatial correlations into account. As a consequence, the pixel-wise differences for metric-guided bounds can be both positive and negative. This indicates that single pixels do not carry sufficient information to explain the variations in dose. We find that the segmentations of the heart are also perceptually different. Pixel-wise upper bounds tend to have larger volumes than lower bounds, while this rule does not hold for metric-guided bounds. Furthermore, this result suggests that pixel-wise and metric-guided methods may disagree on inliers and outliers. Metric-guided inlier reconstructions may have pixels considered as pixel-wise outliers and metric-guided outlier reconstructions may have pixels considered as pixel-wise inliers.

**Figure 3:**
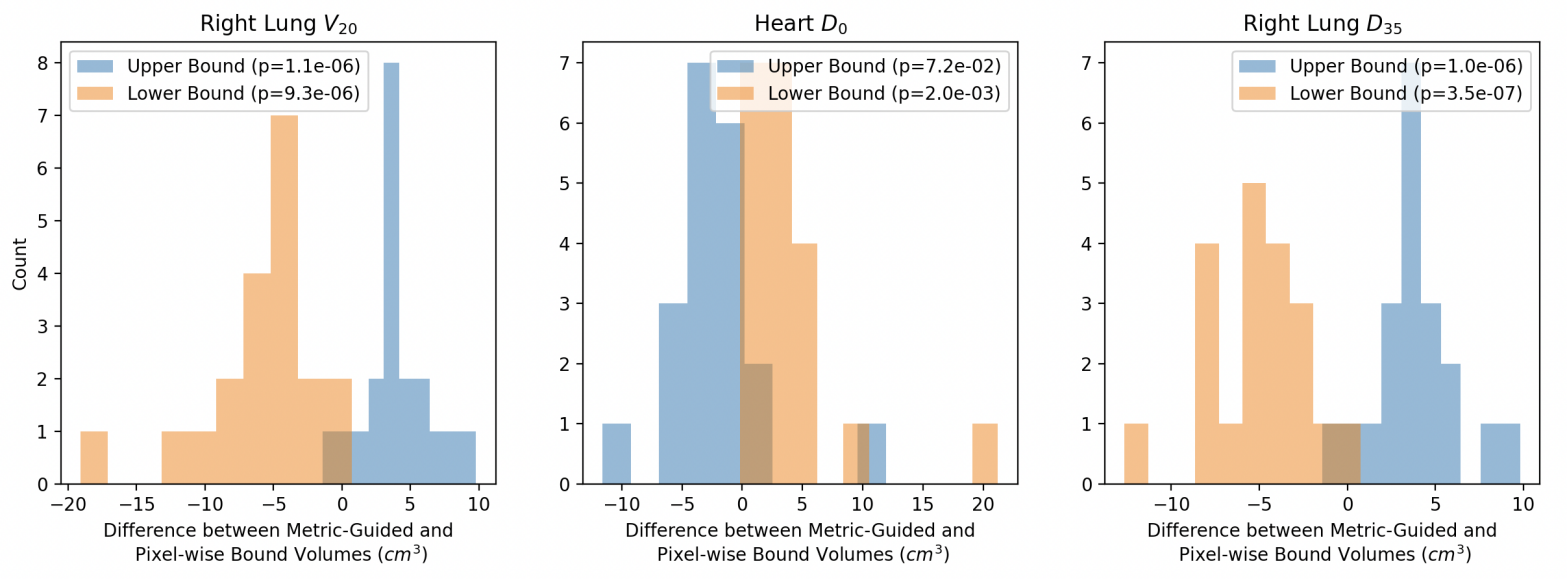
Metric-guided and Pixel-wise methods produce anatomically different upper and lower bounds. We determine whether the upper and lower bound volumes from metric-guided and pixel-wise methods are different across methods using paired t-tests. For all three metrics - volume of ipsilateral lung that received 20Gy (Right Lung $V_{20}$), maximum dose to the heart (Heart $D_{0}$) and dose that 35% volume of the ipsilateral lung receives (Right Lung $D_{35}$), we find that the differences are significant (p<0.05) except for the upper bound reconstructions for Heart $D_{0}$.

Furthermore, we explore the anatomical differences between pixel-wise and metric-guided bounds (Fig. 3). Using organ segmentations from RPA, we determine whether there is a statistically significant difference in upper bound volume across methods and lower bound volume across methods using paired t-tests. We use the dose metrics in Table 1. We find statistically significant differences (p<0.05) for upper and lower bounds across methods except for the upper bound reconstructions for Heart $D_{0}$ (p=7.2e-2) (Fig. 3). This suggests that the upper and lower bounds across methods are anatomically different.

## 5 Conclusion and Discussion

We propose a method that leverages conformal prediction to retrieve upper/lower bounds and statistical inliers/outliers of reconstructions based on the prediction intervals of downstream metrics. We apply our method to sparse CT for downstream radiotherapy planning and show 1) metric-guided bounds have valid coverage for downstream metrics unlike conventional pixel-wise bounds and 2) statistically significant anatomical differences of upper/lower bounds between metric-guided and pixel-wise methods. There are many areas for further investigation:

1. **Factors affecting retrieval error**. Retrieval error may depend on number of samples, the diversity of samples, and the accuracy of the model. We suspect that the larger the number and the diversity of samples, the better the approximation. The prediction intervals and retrieval errors may also be very large if the model is highly biased. Asymmetric bounds could help identify this bias. Furthermore, we assume the downstream processes to be deterministic. This is an appropriate assumption for the maximum dose to the heart, but may not be for other parameters. Opportunities lie in decoupling uncertainty from physical, reconstruction algorithm, and downstream algorithm randomness [37].
2. **Evaluating Safety and Equity**. We can perform patient-specific safety evaluations and identify inequities across patients. For a dose prescription of 50Gy (2Gy/fraction), a safe maximum dose to the heart is <5Gy and the volume of the ipsilateral lung getting 20Gy is <35%. If the upper bound of the prediction interval is greater than these thresholds, it may indicate that the reconstruction is unsuitable for planning. The larger the prediction interval length, the higher the uncertainty. Patients or measurement conditions with high uncertainty can be used for downstream interpretation [38] and action [39, 40]. They may correspond to specific clinical scenarios, such as inadequately filled lungs or large distance from heart to chest wall. Interestingly, we find that pixel-wise volume and intensity intervals for the right lung can explain some of the variance of metric-guided dose intervals for $V_{20}\;\left(R^{2}=0.801\right)$ and $D_{35}\;\left(R^{2}=0.801\right)$. Opportunities lie in applying causal methods [41, 42, 43] to identify factors causes of high uncertainty.
3. **Multiple metrics**. Our method is capable of considering multiple metrics and find reconstructions with all estimated metrics in the prediction intervals containing the ground truth metrics with confidence. While we use a complete set of projections as ground truth, we can also use image quality criteria to assess whether reconstructions meet the measurement standards [44, 45]. Opportunities lie in assessing reconstructions with multiple critical metrics.
4. **Other applications**. Opportunities lie in extending our method to other medical imaging applications [46] and critical scenarios. Additionally, although not demonstrated in our work, our method does not necessitate reconstruction samples to be of identical size or dimensions, as calibration is conducted based on a scalar downstream metric.

## Figures and Tables

**Table 1:** Metric-guided bounds yield valid coverages while conventional pixel-wise bounds do not.

Method	Metric	Avg Test Coverage	Avg Interval Length	Avg P(outlier)

Metric-guided (sym)	Heart *D*_0_ (Gy)	**0.90**	4.39	0.09
Metric-guided (asym)	Heart *D*_0_ (Gy)	**0.90**	37.83	0.09
Pixel-wise	Heart *D*_0_ (Gy)	0.75	0.51	N/A

Metric-guided (sym)	Heart Volume (cm^3^)	**0.90**	166.56	0.03
Metric-guided (asym)	Heart Volume (cm^3^)	**0.90**	531.96	0.01
Pixel-wise	Heart Volume (cm^3^)	0.50	81.46	N/A

Metric-guided (sym)	Right Lung *V*_20_	**0.90**	0.07	0.18
Metric-guided (asym)	Right Lung *V*_20_	**0.90**	0.09	0.17
Pixel-wise	Right Lung *V*_20_	0.50	0.02	N/A

Metric-guided (sym)	Right Lung *D*_35_ (Gy)	**0.90**	9.13	0.14
Metric-guided (asym)	Right Lung *D*_35_ (Gy)	**0.90**	10.25	0.15
Pixel-wise	Right Lung *D*_35_ (Gy)	0.50	1.77	N/A

Metric-guided (sym)	Right Lung Volume (cm^3^)	**0.90**	316.25	0.01
Metric-guided (asym)	Right Lung Volume (cm^3^)	**0.90**	207.27	**0.88**
Pixel-wise	Right Lung Volume (cm^3^)	0.00	0.00	N/A

Using 20 patients and target coverage of 90%, we perform leave-one-out cross-validation and compute average test coverage, interval length, and probability of reconstruction classified as an outlier based on prediction intervals (P(outlier)) using metric-guided (symmetric and asymmetric adjustments) and pixel-wise methods for maximum dose to the heart (Heart *D*_0_), heart volume, volume of ipsilateral lung that received 20Gy (Right Lung *V*_20_), dose that 35% volume of the ipsilateral lung receives (Right Lung *D*_35_), and volume of ipsilateral lung (Right Lung Volume).
